# Molecular diversity of α-gliadin expressed genes in genetically contrasted spelt (*Triticum aestivum* ssp. *spelta*) accessions and comparison with bread wheat (*T. aestivum* ssp. *aestivum*) and related diploid *Triticum* and *Aegilops* species

**DOI:** 10.1007/s11032-016-0569-5

**Published:** 2016-11-10

**Authors:** Benjamin Dubois, Pierre Bertin, Dominique Mingeot

**Affiliations:** 1 Centre wallon de Recherches agronomiques (CRA-W), Département Sciences du vivant, Chaussée de Charleroi, 234, 5030 Gembloux, Belgium; 2 Earth and Life Institute – Agronomy, Université catholique de Louvain (UCL), Croix du Sud, 2 bte L7.05.11, 1348 Louvain-la-Neuve, Belgium

**Keywords:** Spelt, α-gliadin, Celiac disease, Gluten, Genetic diversity

## Abstract

The gluten proteins of cereals such as bread wheat (*Triticum aestivum* ssp. *aestivum*) and spelt (*T. aestivum* ssp. *spelta*) are responsible for celiac disease (CD). The α-gliadins constitute the most immunogenic class of gluten proteins as they include four main T-cell stimulatory epitopes that affect CD patients. Spelt has been less studied than bread wheat and could constitute a source of valuable diversity. The objective of this work was to study the genetic diversity of spelt α-gliadin transcripts and to compare it with those of bread wheat. Genotyping data from 85 spelt accessions obtained with 19 simple sequence repeat (SSR) markers were used to select 11 contrasted accessions, from which 446 full open reading frame α-gliadin genes were cloned and sequenced, which revealed a high allelic diversity. High variations among the accessions were highlighted, in terms of the proportion of α-gliadin sequences from each of the three genomes (A, B and D), and their composition in the four T-cell stimulatory epitopes. An accession from Tajikistan stood out, having a particularly high proportion of α-gliadins from the B genome and a low immunogenic content. Even if no clear separation between spelt and bread wheat sequences was shown, spelt α-gliadins displayed specific features concerning e.g. the frequencies of some amino acid substitutions. Given this observation and the variations in toxicity revealed in the spelt accessions in this study, the high genetic diversity held in spelt germplasm collections could be a valuable resource in the development of safer varieties for CD patients.

**Electronic supplementary material:**

The online version of this article (doi:10.1007/s11032-016-0569-5) contains supplementary material, which is available to authorized users.

## Introduction

Gluten is the result of denaturation of endosperm storage proteins called prolamins during the dough kneading. These prolamins are water-insoluble proteins found in the seeds of bread wheat (*Triticum aestivum* L. ssp. *aestivum*), spelt [*Triticum aestivum* ssp. *spelta* (L.) Thell.] and durum wheat [*Triticum turgidum* ssp. *durum* (Desf.) Husnot]. Equivalent proteins are also found in other cereals, such as barley and rye. Prolamins are composed of monomeric gliadins and polymeric glutenins (Shewry and Halford 2002), which can be divided into high-molecular-weight glutenin subunits (HMW-GS) and low-molecular-weight glutenin subunits (LMW-GS). Conventionally, gliadins are classified into α/β-, γ- and ω-gliadins according to their electrophoretic mobility. Gluten proteins mainly determine the functional properties of wheat flour; glutenins are thought to determine dough elasticity, whereas gliadins could determine its viscosity (Shewry et al. 2003).

The ingestion of gluten peptides can lead to three main types of pathologic reactions: allergic (wheat allergy), autoimmune (celiac disease; CD) and possibly immune-mediated non-celiac gluten sensitivity (NCGS), an unclear grouping of patients whose overall state of health improves when gluten is withdrawn from their diet (Mooney et al. 2013). CD is an uncontrolled inflammatory response to partially digested gluten peptides and is triggered by a T-cell activation in the gastrointestinal mucosa. It results in the flattening of intestinal villi and a reduction in its absorptive capacity, leading to such clinical symptoms as diarrhea, bowel pain, fatigue, weight loss, anemia, osteoporosis, headache and growth retardation (Koning et al. 2005; Marsh 1992; Rashtak and Murray 2012; Sapone et al. 2012). CD affects genetically predisposed individuals, with a prevalence of about 1 % in the human population (Rewers 2005).

Among the gluten proteins, α-gliadins are the most immunogenic fraction with the strongest T-cell activation (Arentz-Hansen et al. 2002; Camarca et al. 2009; Ciccocioppo et al. 2005; Vader et al. 2003). They display four major T-cell stimulatory epitopes: the overlapping DQ2.5-glia-α1 and -α2 epitopes (P{F/Y}PQPQLPY and PQPQLPYPQ, respectively), the DQ2.5-glia-α3 epitope (FRPQQPYPQ) and the DQ8-glia-α1 epitope (QGSFQPSQQ) epitopes. Because the DQ2.5-glia-α2 epitope can be duplicated once or twice, the canonical form of the overlapping DQ2.5-glia-α1 epitope shows two variants: DQ2.5-glia-α1a (PFPQPQLPY) and DQ2.5-glia-α1b (PYPQPQLPY). These four epitopes can be displayed in their canonical form (shown above in brackets), as well as with substituted or deleted amino acid residues. Mitea et al. (2010) showed that these mutations reduce or suppress the antigenic properties of the epitope variants. When two duplications of the DQ2.5-glia-α2 epitope occur, this leads to the full 33-mer fragment, displaying three copies of DQ2.5-glia-α2, which is the most immunogenic fragment of α-gliadin sequences (Molberg et al. 2005; Shan et al. 2002). In addition, α-gliadins display the p31–43 peptide, which induces the innate immune response and enhances the T-cell adaptive response (Gianfrani et al. 2005; Maiuri et al. 2003; Stepniak and Koning 2006).

The α-gliadins constitute the most important class of gliadins as they represent 15–30 % of the bread wheat seed proteins (Gu et al. 2004). Encoded by a multigene family, they possess a very high allelic variability and are located at the Gli-2 loci (Gli-A2, Gli-B2 and Gli-D2) on the short arm of the homeologous chromosomes 6A, 6B and 6D, respectively. The haploid genome includes a number of α-gliadin gene copies ranging from 25 to 35 (Harberd et al. 1985) to 100 (Okita et al. 1985) or even up to 150 (Anderson et al. 1997), depending on the variety.

The development of new cereal varieties that lack immunogenic gluten peptides, but still display good baking properties, constitute one of the new CD therapeutic approaches currently being considered (Rashtak and Murray 2012). It would therefore be relevant to make use of the high variability existing in bread wheat and its related taxa. Among them, spelt could be particularly interesting because of the high genetic diversity held in spelt germplasm collections (An et al. 2005; Bertin et al. 2004; Caballero et al. 2004). In addition, spelt has been less subject to selection pressure than bread wheat. Selection programs, which have focused, inter alia, on the improvement of bread wheat baking qualities, have contributed to a decrease in genetic diversity, especially at the level of α-gliadins and their toxic epitope content (Van den Broeck et al. 2010). Spelt was one of the most important cereals in Europe at the beginning of the twentieth century, but bread wheat almost completely replaced it because of its better baking qualities, higher yields and lower processing costs (Koening et al. 2015). Spelt, however, has several interesting features, such as high vitamin content and nutrition values, robustness, adaptability to soil and climatic conditions, resistance to diseases and nitrogen use efficiency (Caballero et al. 2004; Campbell 1997; Kema 1992). For more than a decade, the popularity of spelt products has been increasing thanks to their pleasant taste and healthy food reputation (Koening et al. 2015; Kozub et al. 2014). Interest in spelt as a crop for organic farming has also increased because of its lower pesticide requirements compared with bread wheat (Kohajdova and Karovicova 2008; Kozub et al. 2014).

Spelt and bread wheat are both allohexaploids (2n = 6× = 42; AABBDD genome), but they seem to have emerged from distinct hybridization events (Dvorak et al. 2012). The origin of spelt is not yet fully understood, but it seems to have emerged in two different places, one in Iran and one in Europe. Iranian spelt might have emerged, like bread wheat, through hybridization between cultivated emmer [*Triticum turgidum* ssp. *dicoccum* (Schrank ex Schübler) Thell., AABB genome] and *Aegilops tauschii* Cosson (DD genome) whereas European spelt could be the result of a cross between cultivated emmer and hexaploid bread wheat (Blatter et al. 2004; Dvorak et al. 2012; Kozub et al. 2014; Salamini et al. 2002).

The objective of this study was to investigate the diversity of α-gliadin expressed genes from spelt compared with bread wheat and diploid species in the Triticeae tribe based on their α-gliadin amino acid sequence composition. The work involved (i) cloning and sequencing full-ORF α-gliadins from genetically contrasted spelt accessions, (ii) studying the allelic variation in the T-cell stimulatory epitopes, (iii) evaluating the toxicity of spelt accessions by analyzing their canonical epitope composition and (iv) comparing spelt sequences to α-gliadins from bread wheat and related diploid *Triticum* and *Aegilops* species in order to find potential spelt specificities.

## Materials and methods

### Genetic diversity analysis and selection of accessions

A working collection of 84 spelt accessions, from 23 countries and 4 continents, has been maintained at the Walloon Agricultural Research Center (CRA-W, Belgium, see Online Resource 1). An Iranian accession (CGN06533, Iran77d), thought to originate from ancestors that differ from those of other spelts (Dvorak et al. 2012), was added to this working collection. The microsatellite data from 19 simple sequence repeat (SSR) markers (Bertin et al. 2004) used on the 85 accessions were subjected to the model-based clustering method implemented with Structure software (v2.3.4; Pritchard et al. 2000) in order to infer the optimal number of groups best describing the population structure. The number of clusters (K) was tested from 1 to 20 with 10 iterations per K, each iteration consisting of 100,000 burn-in steps, followed by 100,000 Markov Chain Monte Carlo (MCMC) repetitions. The admixture ancestry model was chosen and the allele frequencies were assumed to be independent. The ΔK statistics developed by Evanno et al. (2005) was calculated using STRUCTURE HARVESTER software (Earl and vonHoldt 2012). CLUMPP software (Jakobsson and Rosenberg 2007) was used to obtain the mean individual Qmatrix. The log probability [LnP(D)] values calculated with Structure software and the ΔK statistics were used to determine the number of clusters that best described the collection structure. One accession was selected in each cluster (10 in total) for all the experiments described here. Based on the results reported by Dvorak et al. (2012) and on the membership coefficients obtained with CLUMPP software (see below), the Iran77d accession was added to this selection.

### Plant materials

The 11 selected accessions (Table 1) were kindly provided by the United States Department of Agriculture (USDA, Washington, USA), the Vavilov Institute of Plant Genetic Resources (VIR, Saint-Petersburg, Russia) and the Center for Genetic Resources (CGN, Wageningen, The Netherlands). Among these accessions, eight were landraces (BEL08, SPA03, GER11, GER12, TAD06, SWI23, Iran77d and IRA03), two had an uncertain improvement status (DK01 and BUL04) and the final one was breeding material (US06). They were grown in 2014 in field conditions in Belgium and all the immature grains from a self-pollinated ear were harvested 20 days post-anthesis, immediately frozen in liquid nitrogen and stored at −80 °C.

**Table 1:** Total number of pseudogenes and full open reading frames (ORFs) of α-gliadin sequences obtained from 11 contrasted spelt accessions, distribution of the full-ORF sequences and enumeration of the canonical forms of the four T-cell stimulatory epitopes among each genome and each accession.

Name*	**Accession number**	**Total number of sequences**	**Pseudogenes**	**Full ORF**	**A**	**B**	**D**	DQ2.5-glia-α1				DQ2.5-glia-α2				DQ2.5-glia-α3				DQ8-glia-α1			
								A	B	D	TOT	A	B	D	TOT	A	B	D	TOT	A	B	D	TOT
BEL08	PI348315	42	2	40	11	15	14	9	0	16	25	0	0	16	16	9	0	14	23	0	0	3	3
DK01	PI361811	42	0	42	13	19	10	13	0	13	26	0	0	13	13	10	0	7	17	0	0	5	5
SPA03	PI348572	41	1	40	26	8	6	25	0	12	37	0	0	12	12	24	0	6	30	0	0	4	4
BUL04	PI295063	45	2	43	17	15	11	15	0	17	32	0	0	17	17	15	0	9	24	4	0	4	8
GER11	PI348114	41	2	39	13	17	9	13	0	11	24	0	0	11	11	12	0	7	19	1	0	3	4
GER12	PI348120	36	1	35	18	9	8	16	0	10	26	0	0	10	10	16	0	8	24	1	4	2	7
TAD06	K 52437	47	1	46	14	27	5	8	0	7	15	0	0	7	7	14	0	5	19	0	0	1	1
SWI23	PI347939	45	4	41	15	17	9	15	0	12	27	0	0	12	12	15	0	7	22	0	2	2	4
US06	PI355595	38	4	34	21	7	6	14	0	11	25	0	0	11	11	21	0	6	27	0	1	5	6
Iran77d	CGN 06533	45	1	44	22	7	15	12	0	22	34	0	0	22	22	22	0	14	36	0	0	8	8
IRA03	CGN12270	42	0	42	15	11	16	10	0	24	34	0	0	26	26	15	0	10	25	0	0	11	11
		**464**	**18**	**446**	**185**	**152**	**109**	**150**	**0**	**155**	**305**	**0**	**0**	**157**	**157**	**173**	**0**	**93**	**266**	**6**	**7**	**48**	**61**

The 446 full-ORF α-gliadin expressed sequences were checked for the presence of the four T-cell stimulatory epitopes in their canonical forms: P{F/Y}PQPQLPY (DQ2.5-glia-α1), PQPQLPYPQ (DQ2.5-glia-α2), FRPQQPYPQ (DQ2.5-glia-α3) and QGSFQPSQQ (DQ8-glia-α1)

*: Names used for the same accessions in Bertin et al. (2004) except for Iran77d, which was named as in Dvorak et al. (2012)

### mRNA extraction and RT-PCR

For each accession, total RNA was extracted from 100 mg seeds using the NucleoSpin® RNA Plant kit (Macherey-Nagel, Germany). The RNA quality was evaluated by a 1 % agarose gel electrophoresis and the RNA was quantified by spectrometry. First strand cDNA was synthetized from 250 ng RNA with oligo(dT)_18_ primer using the RevertAid H Minus First Strand cDNA Synthesis Kit (Thermo Scientific) in a total volume of 20 μl.

### Cloning and sequencing

The α-gliadin coding sequences were amplified by the specific primers GliFS: 5′-ATGAAGACCTTTCTCATC-3′ and GliRS: 5′-GTTRGTACCGAAGATGCC-3′. The reverse primer is degenerated to work with an α-gliadin panel as wide as possible. Despite this precaution, it should however be noted that this could still lead to the amplification of a subset of expressed α-gliadins and thus to an underestimation of the amount of expressed variants. Moreover, the magnitude of this underestimation could vary from one genome to another. The amplification was carried out in 20 μl reaction volume containing 1.25 U *Pfu* DNA Polymerase (Thermo Scientific), 1 μl cDNA, 2 μl 10× *Pfu* reaction buffer (with 20 mM MgSO_4_), 0.2 mM dNTP, 0.25 μM of each primer and nuclease-free water to reach 20 μl. The polymerase chain reaction (PCR) was performed as described by Mitea et al. (2010).

The PCR products were run on a 1 % agarose gel, purified with the GeneJET Gel Extraction Kit (Thermo Scientific) and cloned in a pJET 1.2/blunt cloning vector using the CloneJET PCR Cloning Kit (Thermo Scientific). Chemical competent cells of the *E. coli* DH5α strain were then transformed and colonies grown after an overnight incubation at 37 °C were checked by colony PCR. Subsequently, the PCR products of about 50 positive clones for each spelt accession were sequenced using the Sanger technique (Beckman Coulter Genomics, United Kingdom).

### Sequence sorting and genome assignment

Given the multigenic character of α-gliadins, there was a risk to obtain chimeric products during the PCR amplification. To avoid it, sequences showing a putative combination of variants were discarded. After withdrawing sequences of poor quality (4), other than α-gliadins (7) or thought to be chimeric (96), the α-gliadin sequences (deposited in GenBank with accession numbers KX173847 through KX174292) were translated into amino acid sequences using BioEdit v7.1.11 (Hall 1999). The identification of nucleic and amino acid sequences present in more than one copy, as well as the elaboration of clusters grouping identical sequences, were carried out with SeqTools v8.4.042 (http://www.seqtools.dk/). This software was also used to search for homologies with all α-gliadin proteins in GenBank via a BLASTP analysis (date of analysis: 23 October 2015).

The amino acid sequences of the four major T-cell stimulatory epitopes (DQ2.5-glia-α1, DQ2.5-glia-α2, DQ2.5-glia-α3 and DQ8-glia-α1) were investigated in order to assign each sequence to a genome, following Van Herpen et al. (2006). For each accession, this attribution was further confirmed by a phylogenetic analysis. The spelt amino acid sequences were first aligned using ClustalW in MEGA6 (Tamura et al. 2013), together with 67 GenBank sequences: 31 from diploid species (15 from *Triticum urartu* Tumanian ex Gandilyan, five from *Aegilops speltoides* Tausch and 11 from *Aegilops tauschii*) and 36 from *T. aestivum* ssp. *aestivum*, previously assigned to one of the three genomes (13 from chromosome 6A, 12 from 6B and 11 from 6D). These sequences are reported in Online Resource 2a. Neighbor-joining trees were then constructed in MEGA6 based on a distance method (Poisson substitution model), with 1000 bootstrap replications, uniform rates among sites, homogeneous pattern among lineages and pairwise deletion of gaps and missing data. In order to compare spelt and bread wheat α-gliadin sequences and to see if spelt characteristics could be pointed out, an overall phylogenetic analysis was conducted in the same way and included all the sequences from the 11 spelt accessions, as well as 210 GenBank α-gliadin sequences from bread wheat and diploid species (see Online Resource 2a and b). The bread wheat sequences included in this analysis came from 31 distinct varieties and corresponded to all bread wheat α-gliadins reported in GenBank after withdrawing pseudogenes and sequences of poor quality.

## Results

### Genetic diversity analysis

**Fig. 1:**
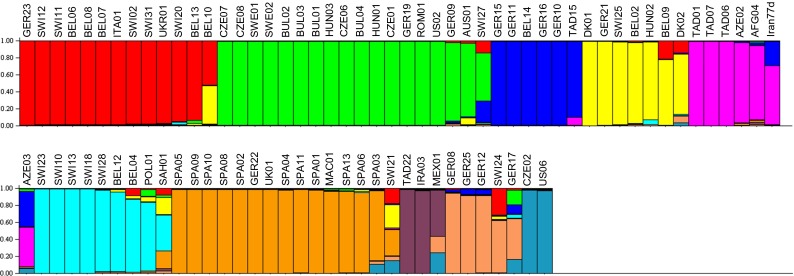
Structure inference in a collection of 85 spelt accessions on the basis of SSR results using Structure software (v2.3.4), clustering for K = 10. Microsatellite data from 19 SSR markers (Bertin et al. 2004) were used to perform this analysis. Each color represents one group and each vertical strip corresponds to one accession. The strips are divided into fragments representing the membership proportion to each group

In order to select contrasted spelt accessions, the model-based clustering method in Structure software was applied to the SSR data of an international collection of 85 spelt accessions. The LnP(D) value calculated by Structure was the highest for K = 10. The ΔK statistics (Evanno et al. 2005) showed two clear peaks at K = 2 and 10. The peak at K = 10 being the highest, 10 was therefore assumed as the number of groups that best described the structure of the spelt collection (Fig. 1).

Among the 85 accessions, 68 (80 %) had a membership coefficient (Q value) higher than 0.9 and the mean Q was equal to 0.91. The composition of some clusters was strongly linked to the geographical provenance of the accessions, e.g. the orange cluster (Fig. 1) that grouped all the Spanish accessions, almost exclusively, and the green cluster that included a high proportion of accessions from Eastern Europe. Nine accessions were clearly admixed (Q < 0.7), including Iran77d. One accession was selected in each of the 10 groups and Iran77d, previously assumed to originate from ancestors that differs from those of the other spelts (Dvorak et al. 2012), was added to this selection.

### Cloning and molecular characterization of spelt α-gliadin genes

In total, 464 α-gliadin expressed sequences were obtained from the 11 spelt accessions, ranging from 36 to 47 sequences per accession (Table 1). Among these, 446 showed a full open reading frame (full ORF). The 18 remaining sequences (3.9 %) had at least one premature stop codon (PSC) and were designated as pseudogenes, in line with previous publications (Noma et al. 2015; Van Herpen et al. 2006; Ozuna et al. 2015).

A clustering was carried out on the 446 full-ORF genes to group the identical sequences. This resulted in 260 and 226 different nucleic and amino acid sequences respectively. The 226 amino acid sequences were analyzed using BLASTP for their homology with all α-gliadins from Triticeae species reported in GenBank. Among them, only 26 showed 100 % homology with other α-gliadins from Triticeae species.

Most α-gliadins have a typical structure (Online Resource 3), starting with an N-terminal signal peptide, followed by a repetitive domain where three types of CD toxic epitopes are located (DQ2.5-glia-α1, -α2 and -α3), two polyglutamine regions separated by a first unique domain and, finally, a second unique domain at the C-terminal side containing a fourth CD epitope (DQ8-glia-α1). With regard to the sequences obtained in this study, all the 446 α-gliadins displayed this classical structure even when one sequence showed a deletion of almost the entire unique domain I (63 amino acids) and an insertion of eight amino acids at the same location (accession number KX173965). Although showing the typical features, 26 sequences displayed an insertion or deletion in at least one domain.

The typical structure of an α-gliadin also contains six cysteine residues: four in the unique domain I and two in the unique domain II (Online Resource 3). Among the 446 sequences, 427 displayed these cysteines. Seventeen of the 19 remaining α-gliadins had a seventh extra cysteine at the beginning of the second unique domain. A loss of cysteine residues was also observed in two sequences: sequence KX173978 showed five cysteines as the result of a C to Y substitution, whereas sequence KX173965 had only two cysteines after the deletion of almost the entire unique domain I.

### Genome assignment and T-cell stimulatory epitope diversity

**Fig. 2:**
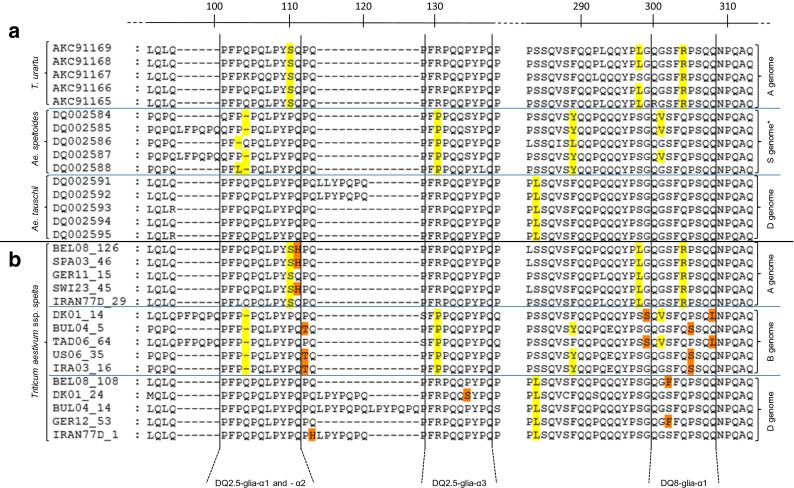
Localization of genome-specific motifs displayed in α-gliadins from (a) diploid species and from (b) spelt. a: For each of the A-, B- and D-genome ancestral species, five α-gliadin sequences selected from GenBank were aligned. b: For each of the three genomes, five representative α-gliadin sequences from spelt obtained in this study were aligned. Yellow residues correspond to genome-specific motifs already identified by Van Herpen et al. (2006) in and around the four major T-cell stimulatory epitopes. Orange residues are new genome-specific motifs discovered in spelt α-gliadins from this study. *: The B genome is hypothesized to be an altered S genome (Von Buren 2001); *Ae. speltoides* is therefore taken as the closest representative of the B genome

In order to assign each sequence to its corresponding genome of origin, we used as reference genome-specific amino acid motifs identified by Van Herpen et al. (2006) in wheat diploid species (highlighted in yellow in Fig. 2a) in and around the four T-cell stimulatory epitopes DQ2.5-glia-α1, -α2, -α3 and DQ8-glia-α1. All these motifs were found in the spelt sequences in this study (highlighted in yellow in Fig. 2b). Some other genome-specific motifs in the same regions, however, were detected in spelt sequences and are highlighted in orange in Fig. 2b.

- DQ2.5-glia-α1 and -α2

The alignment by genomes of spelt sequences allowed three specific motifs to be detected that had not been reported by Van Herpen et al. (2006): (i) a B-genome specific substitution was found at a high frequency (62.9 % of the sequences from the Gli-B2 locus) where the proline amino acid located just after the DQ2.5-glia-α2 epitope was replaced by a threonine (Fig. 2b, amino acid at position 112); (ii) a substitution of the glutamine at the last position (Fig. 2b, p111) of the DQ2.5-glia-α2 epitope by a histidine (PQPQLPYS**H**) occurred in some sequences (14.6 %) of the A genome, but never in the B and D genomes; and (iii) a D-genome specific substitution of a glutamine by a histidine residue at p113 (Fig. 2b) in the second copy of the DQ2.5-glia-α2 epitope (resulting in PQP**H**LPYPQ) was observed in 11 of the 30 sequences displaying one duplication of this epitope.

- DQ2.5-glia-α3

Sequences expressed from the Gli-A2 and Gli-D2 loci are generally not distinguishable from each other because both display the canonical form of the epitope, but we found a substitution of the proline residue at p134 (Fig. 2b) by a serine in 13 (11.9 %) sequences from the D genome (FRPQQ**S**YPQ).

- DQ8-glia-α1

We identified two types into which spelt α-gliadins from the B genome could be divided. The first type was characterized by a tyrosine or sometimes a leucine instead of a phenylalanine residue 11 positions before the epitope (p289 in the Fig. 2), as described by Van Herpen et al. (2006). Remarkably, this mutation was associated with a proline-to-serine substitution at p305 (QGSFQ**S**SQQ) in 89.4 % of the spelt sequences of this type. The second type was characterized by the glycine-to-valine substitution at p301, as reported by Van Herpen et al. (2006). In spelt, this substitution was associated with the replacement of the glutamine residue by a leucine at p308 (Q**V**SFQPSQ**L**) and with a glutamine-to-serine substitution one position before the epitope (p299). Among all the B genome spelt sequences, 73.7 % corresponded to the first type (“YG” type) and 25 % to the second type (“FV” type). Only two α-gliadin sequences from the American accession (US06) could not be classified according to these types because they displayed both a tyrosine residue at p289 and a valine at p301. We also identified in the spelt sequences a high proportion (52.3 %) of α-gliadins from Gli-D2 showing a serine-to-phenylalanine substitution at p302 (QG**F**FQPSQQ).

For each accession, a neighbor-joining tree resulting from the alignment of the amino acid sequences with α-gliadins of known genomes of origin was constructed (data not shown). Three major groups, corresponding to the A, B and D genomes, were clearly displayed in each phylogenetic tree, confirming the genome assignment of the sequences according to the motifs described by Van Herpen et al. (2006).

### Genomic distribution

**Fig. 3:**
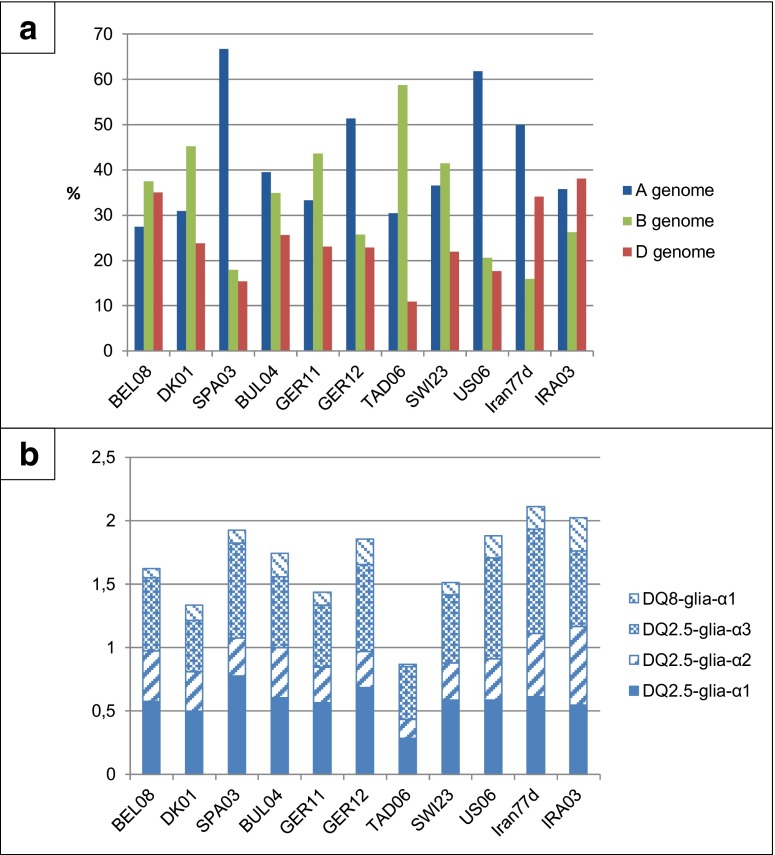
Analysis of α-gliadin transcripts from 11 contrasted spelt accessions: proportion of sequences from the three genomes (a) and average number of canonical epitopes per sequence (b). a: The frequencies were calculated by reporting the number of sequences from each genome to the total number of cloned α-gliadins for each accession. b: Contribution of the four T-cell stimulatory epitopes to the average number of canonical epitopes per sequence

The A, B and D genomes were not equally represented among the 446 full-ORF spelt α-gliadin sequences, with 185 (42 %), 152 (34 %) and 109 (24 %) being counted for the A, B and D genomes, respectively (Table 1). The sequence frequencies in each genome were quite different from one accession to another (Fig. 3a). The Spanish (SPA03) and American (US06) accessions, for example, showed the highest proportion of A genome sequences (more than 60 %), the Tajik accession (TAD06) the highest proportion of B genome sequences (about 60 %) and the Belgian (BEL08) and both Iranian accessions (Iran77d and IRA03) the highest proportion of sequences from the D genome (35–40 %).

Genome-specific variations in the number of glutamine residues in the two polyglutamine regions (PQI and PQII) have been reported several times in *Triticum* and *Aegilops* species (Li et al. 2012; Li et al. 2013; Van Herpen et al. 2006; Xie et al. 2010). In spelt, significant variations in the length of PQI and PQII regions were observed (Online Resource 4). Overall, the PQI region had a higher average number of glutamine residues than the PQII region and displayed a significantly larger average number of glutamine residues in the α-gliadins from the A genome than from the B and D genomes. In contrast, the PQII region had a significantly lower number of Q-residues in the A genome sequences than the B and D genome sequences. The standard deviation of the mean number of Q-residues in the PQII of the B genome sequences was noticeably high.

### Phylogenetic analysis

**Fig. 4:**
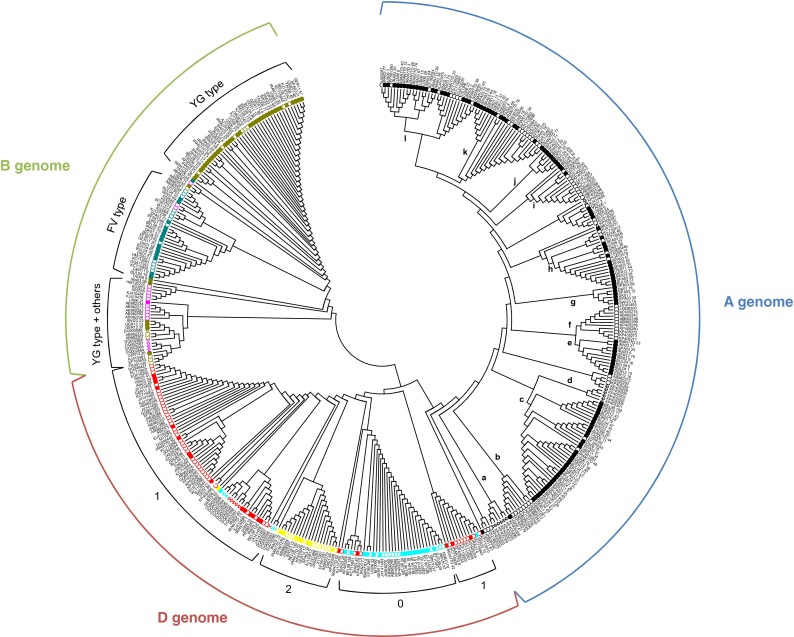
Neighbor-joining tree of 226 spelt α-gliadin amino acid sequences from this study and 210 previously published sequences from diploid species and bread wheat. The neighbor-joining tree is presented in a circular disposition where only the topology is displayed for the sake of clarity. Sequences from diploid species and bread wheat were retrieved from GenBank, and spelt α-gliadins were determined in this study. Alpha-gliadin sequences from diploid species, arising from *Triticum urartu*, *Aegilops speltoides* and *Ae. tauschii*, were labeled by empty triangles, those from bread wheat by empty circles and spelt α-gliadins were labeled by filled circles. Sequences from the A genome were colored black. In the B genome, YG-type and FV-type sequences were colored olive and blue-gray, respectively. Alpha-gliadins matching with neither YG- nor FV-type were colored pink. Spelt sequences from the D genome were marked with a turquoise, red or yellow label when they displayed 0, 1 or 2 duplications of the DQ2.5-glia-α2 epitope, respectively

A phylogenetic analysis was performed involving the 226 different spelt amino acid sequences in this study, 31 α-gliadins from diploid species representing the A, B and D genomes (empty triangles in Fig. 4) and 179 α-gliadins from bread wheat (empty circles). Three main groups were clearly discernible, corresponding to the three genomes, A, B and D.

The A genome cluster was the largest. Even if no clear separation into spelt, bread wheat and *T. urartu* α-gliadins was seen, some sub-clusters were identified, including exclusively or predominantly sequences from spelt (sub-clusters c, e, g, h, j, k, l), bread wheat (b, f, i) or *T. urartu* (a and d).

In the B genome cluster, a grouping coherent with the YG/FV classification was displayed. The sub-cluster at the top of the figure, predominantly composed of spelt sequences, included only YG-type α-gliadins whereas the middle sub-cluster displayed almost only FV-type spelt and bread wheat α-gliadins. The last sub-cluster at the bottom of the B genome cluster included some YG-type spelt α-gliadins but also bread wheat and *Ae. speltoides* α-gliadins that do not match with this YG/FV classification.

In the D genome cluster, sub-groups corresponding to the number of duplication of the DQ2.5-glia-α2 epitope were observed. Both external sub-clusters on the left and the right parts of the cluster displayed mainly α-gliadins with one duplication. Between them, one sub-cluster included α-gliadins with two duplications, i.e. the full 33-mer sequence, whereas the last one contained mainly α-gliadins without duplication. Among this D genome cluster, spelt and bread wheat α-gliadins were rather homogeneously distributed while *Ae. tauschii* sequences seemed to cluster together.

### Canonical epitope screening and inventory of epitope variants

Each of the 446 α-gliadin sequences was manually checked for the presence of the four T-cell stimulatory epitopes DQ2.5-glia-α1, -α2, -α3 and DQ8-glia-α1 in their canonical forms: P{F/Y}PQPQLPY, PQPQLPYPQ, FRPQQPYPQ and QGSFQPSQQ, respectively (Table 1). DQ2.5-glia-α1 and -α3 were the most frequent epitopes, followed by DQ2.5-glia-α2 and finally DQ8-glia-α1. This last-mentioned epitope was the only one that was present in its canonical form in each of the three genomes, whereas the three other intact epitopes were systematically absent from the B genome. The DQ2.5-glia-α2 epitope was always mutated in the A and B genome sequences. The D genome was therefore the only one to display the canonical DQ2.5-glia-α2 epitope, but it was still found in relatively high amounts because of its duplication or triplication in some sequences. These duplications and triplications were displayed in 27.5 % and 13.8 % of the D genome sequences, respectively.

The mean number of canonical epitopes per sequence varied greatly, depending on the accession (Fig. 3b). The spelt from Tajikistan TAD06 was clearly distinct from the others, displaying a mean number of canonical epitopes (0.91) significantly smaller than the average of the 10 remaining ones (1.87). The highest values were observed for the Spanish (SPA03) and two Iranian (Iran77d and IRA03) spelt accessions in relation to the large proportion of sequences from the A and D genome, respectively, found in these accessions.

Variants of the DQ2.5-glia-α1, -α2, -α3 and DQ8-glia-α1 epitopes were also searched in the 446 full-ORF sequences, according to their genome of origin (Online Resource 5). The DQ2.5-glia-α1, -α2 and -α3 epitopes displayed 11, 13 and 8 variants, respectively, and the canonical form was always predominant for all of them. Only mutated variants, however, were observed in sequences from the B genome and the mutations consisted either of a residue deletion (DQ2.5-glia-α1 and -α2) or a residue substitution (DQ2.5-glia-α3). The DQ8-glia-α1 epitope showed the lowest diversity, with seven variants that were always the result of a substitution. Its canonical form was preferentially encountered in the D genome α-gliadins, but remarkably it was not the most frequent form as two other variants, almost exclusively found in the A or B genome, appeared in the first and second positions, respectively.

## Discussion

### Clustering in the spelt collection

The main objective of this study was to investigate the α-gliadin diversity in spelt through the cloning and sequencing of expressed sequences from contrasted spelt accessions. To this end, we started by studying the genetic diversity of a spelt collection. The analysis with Structure software based on 19 SSR markers led to a clustering of 85 accessions in 10 groups broadly coherent with the spelt geographic provenance. This result was consistent with the findings reported by Bertin et al. (2004), where an unweighted pair-group method with arithmetic averaging (UPGMA)-based dendrogram was generated through the calculation of genetic distances (1 – proportion of shared alleles). Based on the high mean membership coefficient (mean Q = 0.91) and the low number of admixed accessions, we assumed that choosing one accession in each of the 10 clusters would provide a panel that was representative of the spelt diversity. Given that Iran77d does not clearly belong to any of the 10 clusters (Q < 0.7) and that it might originate from ancestors that differ from those of the other spelts (Dvorak et al. 2012), it was added to the 10 selected accessions. This panel enabled us to study the diversity of expressed α-gliadin genes of genetically contrasted spelt accessions, to investigate their allelic variations at the level of four major T-cell stimulatory epitopes, to evaluate the toxicity through their canonical epitope composition and to compare these spelt sequences to α-gliadins from bread wheat and related diploid *Triticum* and *Aegilops* species in order to find potential spelt specificities.

### Molecular characterization of α-gliadin expressed sequences

The results obtained in this study revealed a high allelic variation among the α-gliadin expressed sequences cloned from the 11 contrasted accessions. We successfully cloned and sequenced 464 α-gliadin expressed sequences corresponding to 226 different complete amino acid sequences. Among these, only 26 displayed 100 % homology with other α-gliadins from Triticeae species already reported in GenBank. We therefore provided 200 new α-gliadin sequences from spelt; only 44 spelt α-gliadins had previously been reported. Among these 200 new sequences, a rather high number are unique sequences which could mean that only a subset of all expressed α-gliadins has been amplified. This high diversity is consistent with the multigenic character of the α-gliadin family given that several authors have showed that the duplication of α-gliadin genes led to a great gene copy number with high allelic variation (Anderson et al. 1997; Okita et al. 1985).

Among the 464 sequences, 446 displayed a full ORF, whereas only 18 (3.9 %) displayed at least one PSC, and were therefore considered to be pseudogenes. Working on genomic DNA, Anderson and Greene (1997) showed that about half of the α-gliadin genes from bread wheat displayed at least one PSC. More recently, Ozuna et al. (2015) found pseudogene proportions of 39, 76 and 63 % in the genomes of diploid, tetraploid and hexaploid wheat species, respectively. Polyploidization might have contributed to this increase in PSC occurrence because the genetic redundancy created by polyploidy can change the dynamics of coding sequence evolution and lead to the accumulation of PSC in duplicated genes (Akhunov et al. 2013; Mighell et al. 2000). The appearance of a PSC usually results from a C-to-T substitution (18 of the 19 substitutions in this study). As much as 20 % of the total DNA residues can be methylated in plants and a cytidine methylation at the 5-position can lead to an incorrect replication as a thymidine, which favors the C-to-T transition (Anderson and Greene 1997; Gojobori et al. 1982). In this study, cloning the α-gliadin sequences from the transcriptome (cDNA) enabled us to avoid cloning most pseudogenes. The proportion of pseudogenes still observed in the transcript α-gliadins could be explained by the existence of non-functional sequences due to mutations in the protein-coding part while the control elements are maintained, enabling the transcription of the pseudogene (Mighell et al. 2000). The low proportion of sequences displaying PSC (3.9 %) could result from the nonsense-mediated mRNA decay (NMD), which is one of several post-transcriptional mechanisms controlling the quality of mRNA function (Maquat 2004). NMD, also known as mRNA surveillance, eliminates mRNAs displaying PSC in order to prevent the production of potentially deleterious truncated proteins (Maquat 2004; Mitrovich and Anderson 2005).

Although most of the full-ORF spelt sequences in this study displayed the classical α-gliadin structure, 19 sequences had an extra cysteine and two sequences had only two and five cysteines. This could have a direct impact on dough quality because six cysteine residues lead to the formation of three intramolecular disulfide bonds which stabilize the compact globular protein fold, whereas additional cysteines enable intermolecular bonds to be created (Khatkar et al. 2002; Shewry and Tatham 1997). Anderson et al. (2001) and Kasarda (1989) postulated that an odd number of cysteine residues leads to a free cysteine that could participate in gluten polymers.

### T-cell stimulatory epitope diversity

The expression of the four T-cell stimulatory epitopes DQ2.5-glia-α1, -α2, -α3 and DQ8-glia-α1 in the 11 contrasted accessions showed high diversity in terms of quality (canonical or mutated epitope) and quantity. Phylogenetic analyses with α-gliadins from diploid species enabled the spelt α-gliadins to be grouped into three clusters corresponding to the three genomes, as for bread wheat (Van Herpen et al. 2006). We showed that the genome of origin (A, B or D) greatly influenced the α-gliadin immunogenicity in spelt, as reported by Mitea et al. (2010) for bread wheat. The 33-mer, involving six overlapping copies of DQ2.5-glia-α1 and DQ2.5-glia-α2, is the most immunogenic fragment of α-gliadin sequences (Molberg et al. 2005; Shan et al. 2002). Alpha-gliadins with these epitope duplications are D-genome specific and the 33-mer fragment is generally found at a low frequency in bread wheat α-gliadins (Molberg et al. 2005; Ozuna et al. 2015). In this study, we noticed the same trend, with only 13.8 % of the D-genome spelt α-gliadins containing the full 33-mer fragment. In addition, α-gliadin sequences from the D genome were the only ones to display the four canonical epitopes, as already shown in bread wheat and diploid species (Van Herpen et al. 2006). In bread wheat, α-gliadins expressed from the Gli-A2 locus display two canonical epitopes (DQ2.5-glia-α1 and -α3) and a small proportion of them also contain a canonical DQ8-glia-α1 (Li et al. 2012). Mitea et al. (2010) found that there were no canonical HLA-DQ2.5 T-cell epitopes in B genome α-gliadins from bread wheat and that their mutated substitutes did not display any T-cell stimulatory capacity. Given that these features were reflected in the spelt α-gliadin sequences in our study, a high proportion of expressed α-gliadins from the B genome combined with few sequences from the D genome would be desirable in order to develop new spelt varieties with a reduced CD-immunogenic content. Moreover, Mitea et al. (2010) showed that almost all substitutions occurring in the four major T-cell stimulatory epitopes reduced or suppressed their toxicity, regardless of the genome. They synthetized and tested the toxicity of 14- to 17-mer epitope peptides including two variants highlighted in the Fig. 2b: the substitution of the proline residue by a threonine one position after the DQ2.5-glia-α2 (P-PQLPYPQ**T**) and the substitution of the serine at the third position of the DQ8-glia-α1 epitope by a phenylalanine (QG**F**FQPSQQ). Mitea and her colleagues showed that both variants displayed a 1000 times reduced T-cell stimulation compared to the canonical epitope. Such epitope mutations are thus interesting with the aim of lowering the α-gliadin CD-immunogenic content.

Furthermore, we used the mean number of canonical epitopes per α-gliadin sequence as an indicator of the immunogenic content of the 11 spelt accessions and there were great variations, with mean values ranging from 0.87 to 2.11, depending on the accession. Among them, despite a hypothesized difference in phylogenetic origin, the Iran77d accession did not stand out from the others. The Tajik accession TAD06, in relation to the highest proportion of B-genome α-gliadin transcripts, displayed a low mean number of canonical epitopes. Interestingly, several studies have suggested the existence of two independent origins for spelt (one in Europe and one in Asia) and some genetic differences between them have been reported (Blatter et al. 2004; Dvorak et al. 2012; Jaaska 1978). Thus, the reduced immunogenic content of TAD06 and its geographic origin provide an interesting route for exploring the genetic diversity and the α-gliadin composition of spelt accessions originating from Asia.

### Comparison between spelt and bread wheat α-gliadin sequences

The phylogenetic analysis based on the spelt α-gliadin sequences in this study and others from bread wheat and diploid species in the Triticeae tribe did not show a clear separation between them. In the A and B genome, sub-clusters showing preferential grouping were however pointed out. Moreover, significant differences in length in the two polyglutamine regions (PQI and PQII) were observed. These regions play an important role in dough properties because large numbers of glutamine side chains can increase the visco-elasticity properties of dough via intermolecular interactions, given that they are both good hydrogen bond donors and acceptors (Masci et al. 2000). Spelt α-gliadins from the A genome had a PQI region with a significantly higher number of glutamine residues than the PQI regions in the B and D genome sequences. We did not, however, observe spelt α-gliadin sequences from a particular genome with significantly larger PQII regions. This does not accord with the findings of previous studies (Li et al. 2012; Li et al. 2013; Van Herpen et al. 2006; Xie et al. 2010) on Triticeae species other than spelt, showing that the B genome sequences had a significantly higher number of Q-residues in the PQII region. The main reason for such a difference not being observed in these spelt α-gliadins lies in the occurrence of two sub-groups in the B genome sequences, the first one with a low number (6 or 7) and the second one with a large number (from 13 to 25) of Q-residues in the PQII region. Interestingly, we also revealed two sub-groups, YG and FV, in the B genome sequences based on the amino acid patterns located before and in the DQ8-glia-α1 epitope. For 144 out of the 150 B genome sequences (96 %), the YG-type classification was systematically associated with a short PQII region, whereas the FV-type was associated with a long PQII region. In addition, the absence of a clear YG-FV dichotomy in *Ae. speltoides* and bread wheat α-gliadins make them distinguishable from spelt α-gliadins.

A previous study also reported that several bread wheat varieties had fewer α-gliadins expressed from the B genome than from the A and D genomes (Kawaura et al. 2005). Spelt accessions in our study did not display this expression pattern, with the mean proportions of spelt α-gliadin transcripts from A, B and D genomes being 42, 34 and 24 %, respectively, and the B-genome α-gliadin proportion even reaching 59 % in the TAD06 accession. This higher frequency of expressed α-gliadins from the B genome compared with bread wheat suggests that it would be worthwhile paying more attention to spelt in efforts to develop safer varieties for CD patients.

## Electronic supplementary material

ESM 1 (PDF 156 kb)

ESM 2 (PDF 230 kb)

ESM 3 (PDF 130 kb)

ESM 4 (PDF 134 kb)

ESM 5 (PDF 143 kb)

## References

[CR1] Akhunov ED, Sehgal S, Liang H, Wang S, Akhunova AR, Kaur G, Li W, Forrest KL, See D, Simkova H, Ma Y, Hayden MJ, Luo M, Faris JD, Dolezel J, Gill BS (2013). Comparative analysis of syntenic genes in grass genomes reveals accelerated rates of gene structure and coding sequence evolution in polyploid wheat. Plant Physiol.

[CR2] An X, Li Q, Yan Y, Xiao Y, Hsam SLK, Zeller FJ (2005). Genetic diversity of European spelt wheat (*Triticum aestivum* ssp. *spelta* L. Em. Thell.) revealed by glutenin subunit variations at the *Glu-1* and *Glu-3* loci. Euphytica.

[CR3] Anderson OD, Greene FC (1997). The a-gliadin gene family. II. DNA and protein sequence variation, subfamily structure, and origins of pseudogenes. Theor Appl Genet.

[CR4] Anderson OD, Litts JC, Greene FC (1997). The a-gliadin gene family. I. Characterization of ten new wheat a-gliadin genomic clones, evidence for limited sequence conservation of flanking DNA, and southern analysis of the gene family. Theor Appl Genet.

[CR5] Anderson OD, Hsia CC, Torres V (2001). The wheat γ-gliadin genes: characterization of ten new sequences and further understanding of γ-gliadin gene family structure. Theor Appl Genet.

[CR6] Arentz-Hansen EH, McAdam SN, Molberg O, Kristiansen C, Sollid LM (2000). Production of a panel of recombinant gliadins for the characterization of T cell reactivity in coeliac disease. Gut.

[CR7] Arentz-Hansen H, McAdam SN, Molberg O, Fleckenstein B, Lundin KEA, Jorgensen TJD, Jung G, Roepstorff P, Sollid LM (2002). Celiac lesion T cells recognize epitopes that cluster in regions of gliadins rich in proline residues. Gastroenterology.

[CR8] Bertin P, Grégoire D, Massart S, de Froidmont D (2004). High level of genetic diversity among spelt germplasm revealed by microsatellite markers. Genome.

[CR9] Blatter RHE, Jacomet S, Schlumbaum A (2004). About the origin of European spelt (*Triticum spelta* L.): allelic differentiation of the HMW glutenin B1-1 and A1-2 subunit genes. Theor Appl Genet.

[CR10] Caballero L, Martin LM, Alvarez JB (2004). Variation and genetic diversity for gliadins in Spanish spelt wheat accessions. Genet Resour Crop Ev.

[CR11] Camarca A, Anderson RP, Mamone G, Flerro O, Facchiano A, Costantini S, Zanzi D, Sidney J, Auricchio S, Sette A, Troncone R, Gianfrani C (2009). Intestinal T cell responses to gluten peptides are largely heterogeneous: implications for a peptide-based therapy in celiac disease. J Immunol.

[CR12] Campbell KG, Janick J (1997). Spelt: agronomy, genetics, and breeding. Plant Breeding Reviews.

[CR13] Ciccocioppo R, Di Sabatino A, Corazza GR (2005). The immune recognition of gluten in coeliac disease. Clin Exp Immunol.

[CR14] Dvorak J, Deal KR, Luo M-C, You FM, von Borstel K, Dehgani H (2012). The origin of spelt and free-thrishing hexaploid wheat. J Hered.

[CR15] Earl DA, vonHoldt BM (2012). STRUCTURE HARVESTER: a website and program for visualizing STRUCTURE output and implementing the Evanno method. Conserv Genet Resour.

[CR16] Evanno G, Regnaut S, Goudet J (2005). Detecting the number of clusters of individuals using the software STRUCTURE: a simulation study. Mol Ecol.

[CR17] Garcia-Maroto F, Marana C, Garcia-Olmedo F, Carbonero P (1990). Nucleotide sequence of a cDNA encoding an α/β-type gliadin from hexaploid wheat (*Triticum aestivum*). Plant Mol Biol.

[CR18] Gianfrani C, Auricchio S, Troncone R (2005). Adaptative and innate immune responses in celiac disease. Immunol Lett.

[CR19] Gojobori T, Li W-H, Graur D (1982). Patterns of nucleotide substitution in pseudogenes and functional genes. J Mol Evol.

[CR20] Gu YQ, Crossman C, Kong X, Luo M, You FM, Coleman-Derr D, Dubcovsky J, Anderson OD (2004). Genomic organization of the complex α-gliadin gene loci in wheat. Theor Appl Genet.

[CR21] Hall TA (1999). BioEdit: a user-friendly biological sequence alignment editor and analysis program for windows 95/98/NT. Nucl Acids Symp Ser.

[CR22] Harberd NP, Bartels D, Thompson RD (1985). Analysis of the gliadin multigene loci in bread wheat using nullisomic-tetrasomic lines. Mol Gen Genet.

[CR23] Jaaska V (1978). NADP-dependent aromatic alcohol dehydrogenase in polyploid wheats and their diploid relatives. On the origin and phylogeny of polyploid wheats. Theor Appl Genet.

[CR24] Jakobsson M, Rosenberg NA (2007). CLUMPP: a cluster matching and permutation program for dealing with label switching and multimodality in analysis of population structure. Bioinformatics.

[CR25] Kasarda DD (1989) Glutenin structure in relation to wheat quality. In: Wheat is unique. Am Assoc Cereal Chem 277–302

[CR26] Kasarda DD, Okita TW, Bernardin JE, Baecker PA, Nimmo CC, Lew EJ-L, Dietler MD, Greene FC (1984). Nucleic acid (cDNA) and amino acid sequences of α-type gliadins from wheat (*Triticum aestivum*). Proc Natl Acad Sci U S A.

[CR27] Kaur A, Bains NS, Sood A, Yadav B, Sharma P, Kaur S, Garg M, Midha V, Chhuneja P (2016). Molecular characterization of a-gliadin gene sequences in Indian wheat cultivars Vis-à-Vis celiac disease eliciting epitopes. J Plant Biochem Biot.

[CR28] Kawaura K, Mochida K, Ogihara Y (2005). Expression profile of two storage-protein gene families in hexaploid wheat revealed by large-scale analysis of expressed sequence tags. Plant Physiol.

[CR29] Kema GHJ (1992). Resistance in spelt wheat to yellow rust. III. Phylogenetical considerations. Euphytica.

[CR30] Khatkar BS, Fido RJ, Tatham AS, Schofield JD (2002). Functional properties of wheat gliadins. II. Effects on dynamic rheological properties of wheat gluten. J Cereal Sci.

[CR31] Koening A, Konitzer K, Wieser H, Koehler P (2015). Classification of spelt cultivars based on differences in storage protein compositions from wheat. Food Chem.

[CR32] Kohajdova Z, Karovicova J (2008). Nutritional value and baking applications of spelt wheat. Acta Sci Pol Technol Aliment.

[CR33] Koning F, Schuppan D, Cerf-Bensussan N, Sollid LM (2005). Pathomechanisms in celiac disease. Best Pract Res Cl Ga.

[CR34] Kozub NA, Boguslavskii RL, Sozinov IA, Tverdokhleb YV, Xynias IN, Blume YB, Sozinov AA (2014). Alleles at storage protein loci in *Triticum spelta* L. Accessions and their occurrence in related wheats. Cytol Genet.

[CR35] Li J, Wang S, Li S, Ge P, Li X, Ma W, Zeller FJ, Hsam SLK, Yan Y (2012). Variations and classification of toxic epitopes related to celiac disease among α-gliadin genes from four *Aegilops* genomes. Genome.

[CR36] Li J, Wang S-L, Cao M, Lv D-W, Subburaj S, Li X-H, Zeller FJ, Hsam SLK, Yan Y-M (2013). Cloning, expression, and evolutionary analysis of α-gliadin genes from *Triticum* and *Aegilops* genomes. J Appl Genet.

[CR37] Li Y, Xin R, Zhang D, Li S (2014). Molecular characterization of α-gliadin genes from common wheat cultivar Zhengmai 004 and their role in quality and celiac disease. Crop J.

[CR38] Maiuri L, Ciacci C, Ricciardelli I, Vacca L, Raia V, Auricchio S, Picard J, Osman M, Quaratino S, Londei M (2003). Association between innate response to gliadin and activation of pathogenic T cells in coeliac disease. Lancet.

[CR39] Maquat LE (2004). Nonsense-mediated mRNA decay: splicing, translation and mRNP dynamics. Nat Rev Mol Cell Bio.

[CR40] Marsh MN, Marsh MN (1992). Mucosal pathology in gluten sensitivity. Coeliac disease.

[CR41] Masci S, D’Ovidio R, Lafiandra D (2000). A 1B-coded low-molecular-weight glutenin subunit associated with quality in durum wheats shows strong similarity to a subunit present in some bread wheat cultivars. Theor Appl Genet.

[CR42] Mighell AJ, Smith RN, Robinson PA, Markham AF (2000). Vertebrate pseudogenes. FEBS Lett.

[CR43] Mitea C, Salentijn EMJ, van Veelen P, Goryunova SV, van der Meer IM (2010). A universal approach to eliminate antigenic properties of alpha-gliadin peptides in celiac disease. PLoS One.

[CR44] Mitrovich QM, Anderson P (2005). mRNA surveillance of expressed pseudogenes in *C. elegans*. Curr Biol.

[CR45] Molberg O, Uhlen AK, Jensen T, Flaete NS, Fleckenstein B, Arentz-Hansen H, Raki M, Lundin KEA, Sollid LM (2005). Mapping of gluten T-cell epitopes in the bread wheat ancestors: implications for celiac disease. Gastroenterology.

[CR46] Mooney PD, Aziz I, Sanders DS (2013). Non-celiac gluten sensitivity: clinical relevance and recommendations for future research. Neurogastroent Motil.

[CR47] Noma S, Kawaura K, Hayakawa K, Abe C, Tsuge N, Ogihara Y (2015). Comprehensive molecular characterization of the α/β-gliadin multigene family in hexaploid wheat. Mol Gen Genomics.

[CR48] Okita TW, Cheesbrough V, Reeves CD (1985). Evolution and heterogeneity of the α−/β-type and γ-type gliadin DNA sequences. J Biol Chem.

[CR49] Ozuna CV, Iehisa JCM, Gimenez MJ, Alvarez JB, Sousa C, Barro F (2015). Diversification of the celiac disease α-gliadin complex in wheat: a 33-mer peptide with six overlapping epitopes, evolved following polyploidization. Plant J.

[CR50] Pritchard JK, Stephens M, Donnelly P (2000). Inference of population structure using multilocus genotype data. Genetics.

[CR51] Rashtak S, Murray JA (2012). Review article: coeliac disease, new approaches to therapy. Aliment Pharm Ther.

[CR52] Rewers MJ (2005). Epidemiology of celiac disease: what are the prevalence, incidence, and progression of celiac disease?. Gastroenterology.

[CR53] Salamini F, Ozkan H, Brandolini A, Schafer-Pregl R, Martin W (2002). Genetics and geography of wild cereal domestication in the near east. Nat Rev Genet.

[CR54] Sander I, Rozynek P, Rihs H-P, van Kampen V, Chew FT, Lee WS, Kotschy-Lang N, Merget R, Bruning T, Raulf-Heimsoth M (2011). Multiple wheat flour allergens and cross-reactive carbohydrate determinants bind IgE in baker’s asthma. Allergy.

[CR55] Sapone A, Bai JC, Ciacci C, Dolinsek J, Green PHR, Hadjivassiliou M, Kaukinen K, Rostami K, Sanders DS, Schumann M, Ullrich R, Villalta D, Volta U, Catassi C, Fasano A (2012). Spectrum of gluten-related disorders: consensus on new nomenclature and classification. BMC Med.

[CR56] Shan L, Molberg O, Parrot I, Hausch F, Filiz F, Gray GM, Sollid LM, Khosla C (2002). Structural basis for gluten intolerance in celiac sprue. Science.

[CR57] Shewry PR, Halford GH (2002). Cereal seed storage proteins: structures, properties and role in grain utilization. J Exp Bot.

[CR58] Shewry PR, Tatham AS (1997). Disulphide bonds in wheat gluten proteins. J Cereal Sci.

[CR59] Shewry PR, Halford NG, Lafiandra D (2003). Genetics of wheat gluten proteins. Adv Genet.

[CR60] Stepniak D, Koning F (2006). Celiac disease-sandwiched between innate and adaptive immunity. Hum Immunol.

[CR61] Sumner-Smith M, Rafalski JA, Sugiyama T, Stoll M, Söll D (1985). Conservation and variability of wheat α/β-gliadin genes. Nucleic Acids Res.

[CR62] Tamura K, Nei M, Peterson D, Filipski A, Kumar S (2013). MEGA6: molecular evolutionary genetics analysis version 6.0. Mol Biol Evol.

[CR63] Vader LW, Stepniak DT, Bunnik EM, Kooy YMC, De Haan W, Wouter Drijfhout J, Van Veelen PA, Koning F (2003). Characterization of cereal toxicity for celiac disease patients based on protein homology in grains. Gastroenterology.

[CR64] Van den Broeck HC, de Jong HC, Salentijn EMJ, Dekking L, Bosch D, Hamer RJ, Gilissen LJWJ, van der Meer IM, Smulders MJM (2010). Presence of celiac disease epitopes in modern and old hexaploid wheat varieties: wheat breeding may have contributed to increased prevalence of celiac disease. Theor Appl Genet.

[CR65] Van Herpen TWJM, Goryunova SV, van der Schoot J, Mitreva M, Salentijn E, Vorst O, Schenk MF, van Veelen PA, Koning F, van Soest LJM, Vosman B, Bosch D, Hamer RJ, Gilissen LJWJ, Smulders MJM (2006). Alpha-gliadin genes from the a, B and D genomes of wheat contain different sets of celiac disease epitopes. BMC Genomics.

[CR66] Von Buren M (2001). Polymorphisms in two homeologous γ-gliadin genes and the evolution of cultivated wheat. Genet Resour Crop Ev.

[CR67] Xie Z, Wang C, Wang K, Wang S, Li X, Zhang Z, Ma W, Yan Y (2010). Molecular characterization of the celiac disease epitope domains in α-gliadin genes in *Aegilops tauschii* and hexaploid wheats (*Triticum aestivum* L.). Theor Appl Genet.

