# Supplementary material for: Molecular diversity of α-gliadin expressed genes in genetically contrasted spelt (Triticum aestivum ssp. spelta) accessions and comparison with bread wheat (T. aestivum ssp. aestivum) and related diploid Triticum and Aegilops species
Source: Mol Breed. 2016 Nov 10;36(11):152. doi: 10.1007/s11032-016-0569-5 (PMC5104789; doi:10.1007/s11032-016-0569-5)
Supplement: Supplementary file 1 — (PDF 156 kb) [file 11032_2016_569_MOESM1_ESM.pdf]

Molecular diversity of α-gliadin expressed genes in genetically contrasted spelt (*Triticum aestivum* ssp. *spelta)* accessions and comparison with bread wheat (*T. aestivum* ssp. *aestivum)* and related diploid *Triticum* and *Aegilops* species

Molecular Breeding

Benjamin Dubois[1, 2], Pierre Bertin[2], Dominique Mingeot[1]

[1] Centre wallon de Recherches agronomiques (CRA-W), Département Sciences du vivant, Chaussée de Charleroi, 234, 5030 Gembloux, Belgium

[2] Université catholique de Louvain (UCL), Earth and Life Institute – Agronomy, Croix du Sud, 2 bte L7.05.11, 1348 Louvain-la-Neuve, Belgium

Corresponding author: Benjamin Dubois, b.dubois@cra.wallonie.be

Online Resource 1. Passport data of the 85 spelt accessions submitted to a genetic assignment method analysis with Structure software.

Provenance area	Provenance country	Name [1]	Accession number	Accession Name	Germplasm [2]
Africa	Western Sahara	SAH01	CGN08284	Sahara I	CGN (Wageningen, The Netherlands)
America	Mexico	MEX01	PI520066	26867-302y-300m-oy	USDA (Washington, USA)
	USA	US02	PI168681	White spring	USDA (Washington, USA)
		US06	PI355595	69Z5,73	USDA (Washington, USA)
Eastern Europe	Bulgaria	BUL01	PI295059	Deutchland	USDA (Washington, USA)
		BUL02	PI295061	Ungarn	USDA (Washington, USA)
		BUL03	PI295062	Italien	USDA (Washington, USA)
		BUL04	PI295063	Ungarn	USDA (Washington, USA)
	Czech Republic	CZE01	RICP 01c0100920	(svetla)	CRI (Prague, Czech Republic)
		CZE02	RICP 01c0100921	(tmava)	CRI (Prague, Czech Republic)
		CZE06	RICP 01c0200982	spalda bila jarni	CRI (Prague, Czech Republic)
		CZE07	RICP 01c0200983		CRI (Prague, Czech Republic)
		CZE08	RICP 01c0201257	(ruzyne)	CRI (Prague, Czech Republic)
	Hungary	HUN01	PI272574	I-1-3540	USDA (Washington, USA)
		HUN02	PI272579	I-1-3530	USDA (Washington, USA)
		HUN03	PI290514	White spelt	USDA (Washington, USA)
	Macedonia	MAC01	PI378469	1744	USDA (Washington, USA)
	Poland	POL01	PI192717	Dankowska graniatka	USDA (Washington, USA)
	Romania	ROM01	PI306554	2947	USDA (Washington, USA)
	Ukraine	UKR01	K 19372		VIR (Saint-Petersburg, Russia)
Northwestern Europe	Austria	AUS01		Ebners rotkorn	
	Belgium	BEL02		Franckenkorn	CRA-W (Gembloux, Belgium)
		BEL04		Redouté	CRA-W (Gembloux, Belgium)
		BEL06	PI348303	69Z6,472	USDA (Washington, USA)
		BEL07	PI348312	69Z6,482	USDA (Washington, USA)
		BEL08	PI348315	69Z6,485	USDA (Washington, USA)
		BEL09	PI348329	69Z6,499	USDA (Washington, USA)
		BEL10	PI348334	69Z6,505	USDA (Washington, USA)
		BEL12	PI348366	69Z6,538	USDA (Washington, USA)
		BEL13	PI348409	69Z6,582	USDA (Washington, USA)
		BEL14	PI348417	69Z6,590	USDA (Washington, USA)
	Denmark	DK01	PI361811	DN-2267	USDA (Washington, USA)
		DK02	PI361812	DN-2268	USDA (Washington, USA)
	Germany	GER08	PI348033	69Z6,191	USDA (Washington, USA)
		GER09	PI348040	69Z6,198	USDA (Washington, USA)

		GER10	PI348056	69Z6,215	USDA (Washington, USA)
		GER11	PI348114	69Z6,275	USDA (Washington, USA)
		GER12	PI348120	69Z6,282	USDA (Washington, USA)
		GER15	PI348159	69Z6,322	USDA (Washington, USA)
		GER16	PI348168	69Z6,332	USDA (Washington, USA)
		GER17	PI348171	69Z6,335	USDA (Washington, USA)
		GER19	PI355552	T2	USDA (Washington, USA)
		GER21	PI355677	BP 2	USDA (Washington, USA)
		GER22	TRI3445	Blauer Samtiger	IPK (Gatersleben, Germany)
		GER23	TRI1259	Rottweiler Frûhkorn St. I	IPK (Gatersleben, Germany)
		GER25	TRI 303	Kipperhaus Weisser Spelz	IPK (Gatersleben, Germany)
	Sweden	SWE01	NGB 4495	Speltvete FR Gotland	NGB (Alnarp, Sweden)
		SWE02	CGN08300		CGN (Wageningen, The Netherlands)
	Switzerland	SWI02		Balmegg	
		SWI10	RAC TS2113	SCHNOTTWILER WEISSKORN 35	Agroscope Changins (Nyon, Switzerland)
		SWI11	RAC TS2145	Thuerig rotkorn Th4	Agroscope Changins (Nyon, Switzerland)
		SWI12	RAC TS2144	Hueslen rotkorn	Agroscope Changins (Nyon, Switzerland)
		SWI13	RAC TS2117	Neuegger weisskorn Ngg42	Agroscope Changins (Nyon, Switzerland)
		SWI18	RAC TS2116	Willisauer weisskorn Wil17	Agroscope Changins (Nyon, Switzerland)
		SWI20	PI347904	69Z6,57	USDA (Washington, USA)
		SWI21	PI347913	69Z6,66	USDA (Washington, USA)
		SWI23	PI347939	69Z6,93	USDA (Washington, USA)
		SWI24	PI347950	69Z6,105	USDA (Washington, USA)
		SWI25	PI348004	69Z6,161	USDA (Washington, USA)
		SWI27	PI355560	SK1B	USDA (Washington, USA)
		SWI28	PI355573	SK3F	USDA (Washington, USA)
		SWI31	PI355653	Rottweiller Fruhkorn 4	USDA (Washington, USA)
	United Kingdom	UK01	PI355704	69Z5.194	USDA (Washington, USA)
Southwestern	Italy	ITA01	PI355642	69Z5,122	USDA (Washington, USA)
Europe	Spain	SPA01	PI348526	69Z6,704	USDA (Washington, USA)
		SPA02	PI348537	69Z6,715	USDA (Washington, USA)
		SPA03	PI348572	69Z6,752	USDA (Washington, USA)
		SPA04	PI348580	69Z6,760	USDA (Washington, USA)
		SPA05	PI348588	69Z6,768	USDA (Washington, USA)
		SPA06	PI348651	69Z6,834	USDA (Washington, USA)
		SPA08	PI348688	69Z6,872	USDA (Washington, USA)
		SPA09	PI348716	69Z6,900	USDA (Washington, USA)
		SPA10	PI348723	69Z6,908	USDA (Washington, USA)
		SPA11	PI348766	69Z6,953	USDA (Washington, USA)
		SPA13	PI348463	69Z6,638	USDA (Washington, USA)
Middle East	Afghanistan	AFG04	PI367202	625	USDA (Washington, USA)
	Tajikistan	TAD01	K 52443		VIR (Saint-Petersburg, Russia)
		TAD06	K 52437		VIR (Saint-Petersburg, Russia)
		TAD07	K 52442		VIR (Saint-Petersburg, Russia)
		TAD15	K 52463		VIR (Saint-Petersburg, Russia)
		TAD22	K 56568		VIR (Saint-Petersburg, Russia)
Near East	Azerbaijan	AZE02	K 45364		VIR (Saint-Petersburg, Russia)
		AZE03	K 45368		VIR (Saint-Petersburg, Russia)
	Iran	IRA03	CGN12270	Iran 416A	CGN (Wageningen, The Netherlands)
		Iran77d	CGN06533	Iran 77d	USDA (Washington, USA)

[1] : Names used for the same accessions in Bertin et al. (2004) except for Iran77d, which was named as in Dvorak et al. (2012).

[2] : USDA = United States Department of Agriculture; VIR = Vavilov Institute of Plant Genetic Resources; CRI = Crop Research Institute;

IPK = Institute of Plant Genetics and Crop Plant Research; CGN = Center for Genetic Resources; CRA-W = Centre wallon de Recherches agronomiques; NGB = Nordic Gene Bank.
