# Supplementary material for: Molecular diversity of α-gliadin expressed genes in genetically contrasted spelt (Triticum aestivum ssp. spelta) accessions and comparison with bread wheat (T. aestivum ssp. aestivum) and related diploid Triticum and Aegilops species
Source: Mol Breed. 2016 Nov 10;36(11):152. doi: 10.1007/s11032-016-0569-5 (PMC5104789; doi:10.1007/s11032-016-0569-5)
Supplement: Supplementary file 2 — (PDF 230 kb) [file 11032_2016_569_MOESM2_ESM.pdf]

Molecular diversity of α-gliadin expressed genes in genetically contrasted spelt (*Triticum aestivum* ssp. *spelta)* accessions and comparison with bread wheat (*T. aestivum* ssp. *aestivum)* and related diploid *Triticum* and *Aegilops* species

Molecular Breeding

Benjamin Dubois[1, 2], Pierre Bertin[2], Dominique Mingeot[1]

[1] Centre wallon de Recherches agronomiques (CRA-W), Département Sciences du vivant,

Chaussée de Charleroi, 234, 5030 Gembloux, Belgium

[2] Université catholique de Louvain (UCL), Earth and Life Institute – Agronomy,

Croix du Sud, 2 bte L7.05.11, 1348 Louvain-la-Neuve, Belgium

Corresponding author: Benjamin Dubois, b.dubois@cra.wallonie.be

Online Resource 2. (a) List of α-gliadin sequences from ancestral diploid species and from bread wheat used to confirm the genome assignments. (b) List of additional α-gliadin sequences from bread wheat used to conduct an overall phylogenetic analysis together with α-gliadin sequences from bread wheat and diploid species included in the Online Resource 2a and with spelt α-gliadins isolated in this work.

a			
**Genome**	**Species**	**Accession number**	**Reference**
A	*T. urartu*	KP405293, KP405294, KP405295, KP405296, KP405297, KP405298, KP405299, KP405301, KP405302, KP405303, KP405304, KP405305, KP405306, KP405307, KP405308	Ozuna et al. 2015
	var. Cheyenne	U08287	Blechl and Anderson 1994*
	T. aestivum ssp. *aestivum* var. Cheyenne	U50984, U51306, U51307	Anderson et al. 1997
	var. Chinese Spring	AB982242, AB982245, AB982249, AB982255, AB982272, AB982277, AB982278, AB982281, AB982288	Noma et al. 2015
S/B	*Ae. speltoides*	DQ002584, DQ002585, DQ002586, DQ002587, DQ002588	Van Herpen et al. 2006
	var. Cheyenne	K02068	Kasarda et al. 1984
	var. Cheyenne	M11073	Okita et al. 1985
	T. aestivum ssp. *aestivum* var. Cheyenne	U51303	Anderson et al. 1997
		X02540	Sumner-Smith et al. 1985
	var. Chinese Spring	AB982234, AB982236, AB982237, AB982241, AB982267, AB982273, AB982285, AB982286	Noma et al. 2015
D	*Ae. tauschii*	DQ002589, DQ002590, DQ002591, DQ002592, DQ002593, DQ002594, DQ002595, DQ002596, DQ002597, DQ002598, DQ002599	Van Herpen et al. 2006
	var. Cheyenne	M11075	Okita et al. 1985
	T. aestivum ssp. *aestivum* var. Chinese Spring	X17361	Garcia-Maroto et al. 1990
	var. Chinese Spring	AB982248, AB982253, AB982256, AB982260, AB982265, AB982276, AB982279, AB982284, AB982293	Noma et al. 2015

b			
	T. aestivum ssp. *aestivum* var. Mjoelner	AJ133602, AJ133604, AJ133605, AJ133612	Arentz-Hansen et al. 2000
	T. aestivum ssp. *aestivum* var. Jimai20	JN831386	Li and Li 2011*
	T. aestivum ssp. *aestivum* var. Zhengmai9023	KC715927, KC715929, KC715930, KC715932, KC715940, KC715941, KC715944, KC715945, KC715950, KC715951, KC715952, KC715953	Li et al. 2013*
	T. aestivum ssp. *aestivum* var. Zhengmai9023	JX828270	Li et al. 2012*
	T. aestivum ssp. *aestivum* var. C273	KJ410475	Kaur et al.2016
	T. aestivum ssp. *aestivum* var. GW273	KJ410476	Kaur et al.2016
	T. aestivum ssp. *aestivum* var. HI617	KJ410477	Kaur et al.2016

A, B, D	*T. aestivum* ssp. *aestivum* var. Hyb65	KJ410478	Kaur et al.2016
	T. aestivum ssp. *aestivum* var. Lok1	KJ410481	Kaur et al.2016
	T. aestivum ssp. *aestivum* var. NP4	KJ410483	Kaur et al.2016
	T. aestivum ssp. *aestivum* var. NP824	KJ410484	Kaur et al.2016
	T. aestivum ssp. *aestivum* var. VL404	KJ410486	Kaur et al.2016
	T. aestivum ssp. *aestivum* var. WG377	KJ410487	Kaur et al.2016
	T. aestivum ssp. *aestivum* var. WL711	KJ410488	Kaur et al.2016
	T. aestivum ssp. *aestivum* var. Zhengmai004	KC715889, KC715890, KC715892, KC715896, KC715897, KC715901, KC715906, KC715907, KC715908, KC715910, KC715913, KC715917, KC715920, HM120221, HM120222	Li et al. 2014
	T. aestivum ssp. *aestivum* var. Yamhill	K03074, K03075, K03076	Sumner-Smith et al. 1985
	T. aestivum ssp. *aestivum* var. Wyuna	FN391140	Sander et al. 2011
	T. aestivum ssp. *aestivum* var. Gaocheng 8901	EF561274, EF561275, EF561276, EF561278, EF561279, EF561280	Xie et al. 2010
	T. aestivum ssp. *aestivum* var. Zhongyou 9507	EF561281, EF561283, EF561285, EF561287, EF561288	Xie et al. 2010
	T. aestivum ssp. *aestivum* var. AS1643	DQ166376	Chen et al. 2005*
	T. aestivum ssp. *aestivum* var. Jinan177	EU018291, EU018292, EU018295, EU018296, EU018297, EU018298, EU018299	Chen et al. 2007*
	T. aestivum ssp. *aestivum* var. Shaan253	GQ891683, GQ891685	Li et al. 2009*
	T. aestivum ssp. *aestivum* var. Chinese Spring	JX141483, JX141484, JX141485, JX141486, JX141488, JX141489, JX141490, JX141492, JX141493, JX141494, JX141495	Li et al. 2012*
	T. aestivum ssp. *aestivum* var. Yumai50	JX828260	Li et al. 2012*
	T. aestivum ssp. *aestivum* var. Zhengfeng5	JX828284, JX828286, JX828291, JX828292, JX828293, JX828296, JX828298, JX828299, JX828300, JX828304, JX828306, JX828307, JX828310	Li et al. 2012*
	T. aestivum ssp. *aestivum* var. Zhengmai366	JX828318	Li et al. 2012*
	T. aestivum ssp. *aestivum*	DQ417343	Liu et al. 2006*
	T. aestivum ssp. *aestivum* var. Chuannong 16	DQ246446, DQ246447, DQ246448	Liu et al. 2005*
	T. aestivum ssp. *aestivum* var. Chinese Spring	KJ137236, KJ137239, KJ137240, KJ137242, KJ137243	Liu et al. 2014*
	T. aestivum ssp. *aestivum* var. Chinese Spring	AB982235, AB982282, AB982291, AB982292, AB982294, AB982296, AB982298, AB982299	Noma et al. 2015
	T. aestivum ssp. *aestivum* var. Cheyenne	M10092	Okita et al. 1985
	T. aestivum ssp. *aestivum* var. Conil	KP405248, KP405249, KP405252, KP405254, KP405255, KP405260, KP405261, KP405262, KP405263, KP405266, KP405267, KP405268, KP405269, KP405270, KP405271, KP405272, KP405273, KP405275, KP405276, KP405277, KP405278, KP405279, KP405280, KP405281, KP405282	Ozuna et al. 2015
	T. aestivum ssp. *aestivum*	X02538, X02539	Sumner-Smith et al. 1985
	T. aestivum ssp. *aestivum* var. 96-212-2	EF165553, EF165556	Tang et al. 2006*
	T. aestivum ssp. *aestivum* var. 96-132-1	EF165554	Tang et al. 2006*
	T. aestivum ssp. *aestivum* var. 96-137-3	EF165555	Tang et al. 2006*

References :

*: Direct submission

Anderson OD, Litts JC and Greene FC (1997) The a-gliadin gene family. I. Characterization of ten new wheat a-gliadin genomic clones, evidence for limited sequence conservation of flanking DNA, and Southern analysis of the gene family. Theor Appl Genet 95:50-58. doi: 10.1007/s001220050531

Arentz-Hansen EH, McAdam SN, Molberg O, Kristiansen C and Sollid LM (2000) Production of a panel of recombinant gliadins for the characterization of T cell reactivity in coeliac disease. Gut 46:46-51. doi: 10.1136/gut.46.1.46

Garcia-Maroto F, Marana C, Garcia-Olmedo F and Carbonero P (1990) Nucleotide sequence of a cDNA encoding an α/β-type gliadin from hexaploid wheat (*Triticum aestivum*). Plant Mol Biol 14:867-868. doi: 10.1007/BF00016521

Kasarda DD, Okita TW, Bernardin JE, Baecker PA, Nimmo CC, Lew EJ-L, Dietler MD and Greene FC (1984) Nucleic acid (cDNA) and amino acid sequences of α-type gliadins from wheat (*Triticum aestivum*). Proc Natl Acad Sci USA 81:4712-4716.

Kaur A, Bains NS, Sood A, Yadav B, Sharma P, Kaur S, Garg M, Midha V and Chhuneja P (2016) Molecular characterization of a-gliadin gene sequences in Indian wheat cultivars vis-à-vis celiac disease eliciting epitopes. J Plant Biochem Biot. doi: 10.1007/s13562-016-0367-5

Li Y, Xin R, Zhang D and Li S (2014) Molecular characterization of α-gliadin genes from common wheat cultivar Zhengmai 004 and their role in quality and celiac disease. Crop J 2:10-21. doi: 10.1016/j.cj.2013.11.003

Noma S, Kawaura K, Hayakawa K, Abe C, Tsuge N and Ogihara Y (2015) Comprehensive molecular characterization of the α/β-gliadin multigene family in hexaploid wheat. Mol Genet Genomics 291:65-77. doi: 10.1007/s00438-015-1086-7

Okita TW, Cheesbrough V and Reeves CD (1985) Evolution and heterogeneity of the α-/β-type and γ-type gliadin DNA sequences. J Biol Chem 260:8203-8213.

Ozuna CV, Iehisa JCM, Gimenez MJ, Alvarez JB, Sousa C and Barro F (2015) Diversification of the celiac disease α-gliadin complex in wheat: a 33-mer peptide with six overlapping epitopes, evolved following polyploidization. Plant J 82:794-805. doi: 10.1111/tpj.12851

Sander I, Rozynek P, Rihs H-P, van Kampen V, Chew FT, Lee WS, Kotschy-Lang N, Merget R, Bruning T and Raulf-Heimsoth M (2011) Multiple wheat flour allergens and cross-reactive carbohydrate determinants bind IgE in baker’s asthma. Allergy 66:1208-1215. doi: 10.1111/j.1398-9995.2011.02636.x

Sumner-Smith M, Rafalski JA, Sugiyama T, Stoll M and Söll D (1985) Conservation and variability of wheat α/β-gliadin genes. Nucleic Acids Res 13:3905-3916. doi: 10.1093/nar/13.11.3905

Van Herpen TWJM, Goryunova SV, van der Schoot J, Mitreva M, Salentijn E, Vorst O, Schenk MF, van Veelen PA, Koning F, van Soest LJM, Vosman B, Bosch D, Hamer RJ, Gilissen LJWJ and Smulders MJM (2006) Alpha-gliadin genes from the A, B and D genomes of wheat contain different sets of celiac disease epitopes. BMC Genomics 7:1. doi: 10.1186/1471-2164-7-1

Xie Z, Wang C, Wang K, Wang S, Li X, Zhang Z, Ma W and Yan Y (2010) Molecular characterization of the celiac disease epitope domains in α-gliadin genes in *Aegilops tauschii* and hexaploid wheats (*Triticum aestivum* L.). Theor Appl Genet 121:1239-1251. doi: 10.1007/s00122-010-1384-8
