# Supplementary material for: Molecular diversity of α-gliadin expressed genes in genetically contrasted spelt (Triticum aestivum ssp. spelta) accessions and comparison with bread wheat (T. aestivum ssp. aestivum) and related diploid Triticum and Aegilops species
Source: Mol Breed. 2016 Nov 10;36(11):152. doi: 10.1007/s11032-016-0569-5 (PMC5104789; doi:10.1007/s11032-016-0569-5)

Molecular diversity of α-gliadin expressed genes in genetically contrasted spelt (*Triticum aestivum* ssp. *spelta)* accessions and comparison with bread wheat (*T. aestivum* ssp. *aestivum)* and related diploid *Triticum* and *Aegilops* species

Molecular Breeding

Benjamin Dubois[1, 2], Pierre Bertin[2], Dominique Mingeot[1]

[1] Centre wallon de Recherches agronomiques (CRA-W), Département Sciences du vivant, Chaussée de Charleroi, 234, 5030 Gembloux, Belgium

[2] Université catholique de Louvain (UCL), Earth and Life Institute – Agronomy, Croix du Sud, 2 bte L7.05.11, 1348 Louvain-la-Neuve, Belgium

Corresponding author: Benjamin Dubois, b.dubois@cra.wallonie.be

Online Resource 3. Typical structure of an α-gliadin. The four major T-cell stimulatory epitopes and their corresponding amino acid sequences are represented below the polypeptide model. 'C' indicates the classical location of the cysteine residues

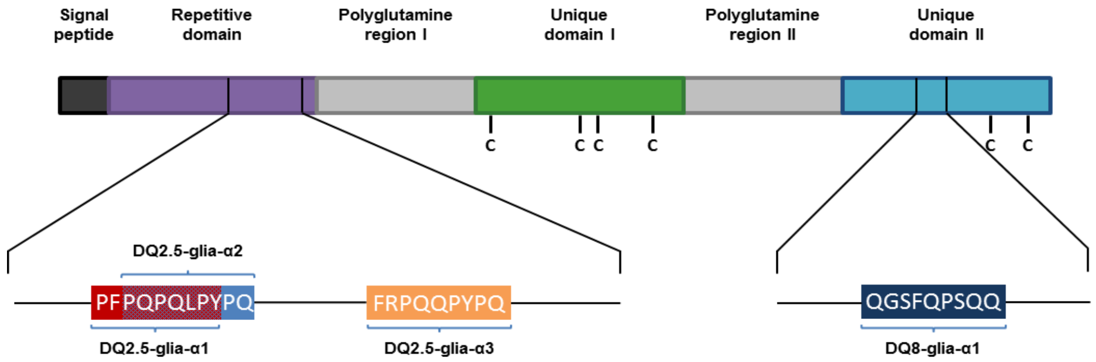

Supplement: Supplementary file 3 — (PDF 130 kb) [file 11032_2016_569_MOESM3_ESM.pdf]
