# Supplementary material for: Molecular diversity of α-gliadin expressed genes in genetically contrasted spelt (Triticum aestivum ssp. spelta) accessions and comparison with bread wheat (T. aestivum ssp. aestivum) and related diploid Triticum and Aegilops species
Source: Mol Breed. 2016 Nov 10;36(11):152. doi: 10.1007/s11032-016-0569-5 (PMC5104789; doi:10.1007/s11032-016-0569-5)

Molecular diversity of α-gliadin expressed genes in genetically contrasted spelt (*Triticum aestivum* ssp. *spelta)* accessions and comparison with bread wheat (*T. aestivum* ssp. *aestivum)* and related diploid *Triticum* and *Aegilops* species

Molecular Breeding

Benjamin Dubois[1, 2], Pierre Bertin[2], Dominique Mingeot[1]

[1] Centre wallon de Recherches agronomiques (CRA-W), Département Sciences du vivant, Chaussée de Charleroi, 234, 5030 Gembloux, Belgium

[2] Université catholique de Louvain (UCL), Earth and Life Institute – Agronomy, Croix du Sud, 2 bte L7.05.11, 1348 Louvain-la-Neuve, Belgium

Corresponding author: Benjamin Dubois, b.dubois@cra.wallonie.be

Online Resource 4. Average number of glutamine residues in the first (PQI) and second (PQII) polyglutamine regions in 226 α-gliadin transcripts from 11 spelt accessions, according to the genome of origin (A, B and D). The PQI region overall had a higher average number of glutamine residues (16.8 +/- 4.1) than the PQII region (9.0 +/- 4.4). Alpha-gliadins from the A genome displayed a significantly higher average number of glutamine residues in the PQI region (19.3 +/- 3.1) than B (13.5 +/- 3.5) and D (14.6 +/- 2.9) genome sequences. For the PQII region, A genome sequences had a significantly lower average number of glutamine residues (6.9 +/- 0.5) than B (11.1 +/- 7.3) and D (11.6 +/- 2.2) genome α-gliadins.

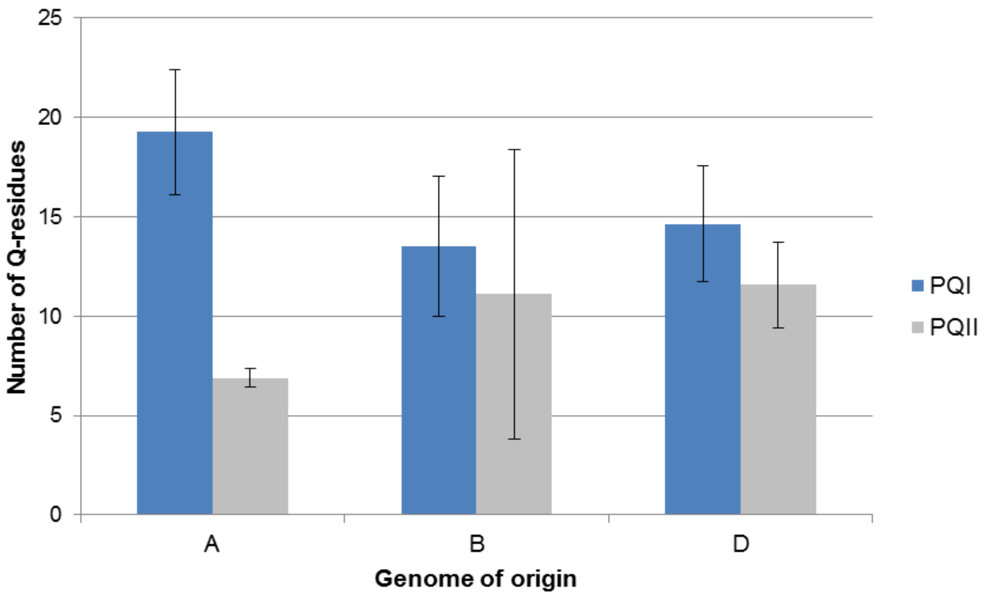

Supplement: Supplementary file 4 — (PDF 134 kb) [file 11032_2016_569_MOESM4_ESM.pdf]
