# Supplementary material for: Molecular diversity of α-gliadin expressed genes in genetically contrasted spelt (Triticum aestivum ssp. spelta) accessions and comparison with bread wheat (T. aestivum ssp. aestivum) and related diploid Triticum and Aegilops species
Source: Mol Breed. 2016 Nov 10;36(11):152. doi: 10.1007/s11032-016-0569-5 (PMC5104789; doi:10.1007/s11032-016-0569-5)
Supplement: Supplementary file 5 — (PDF 143 kb) [file 11032_2016_569_MOESM5_ESM.pdf]

Molecular diversity of α-gliadin expressed genes in genetically contrasted spelt (*Triticum aestivum* ssp. *spelta)* accessions and comparison with bread wheat (*T. aestivum* ssp. *aestivum)* and related diploid *Triticum* and *Aegilops* species

Molecular Breeding

Benjamin Dubois[1, 2], Pierre Bertin[2], Dominique Mingeot[1]

[1] Centre wallon de Recherches agronomiques (CRA-W), Département Sciences du vivant,

Chaussée de Charleroi, 234, 5030 Gembloux, Belgium

[2] Université catholique de Louvain (UCL), Earth and Life Institute – Agronomy,

Croix du Sud, 2 bte L7.05.11, 1348 Louvain-la-Neuve, Belgium

Corresponding author: Benjamin Dubois, b.dubois@cra.wallonie.be

Online Resource 5. Variants of the DQ2.5-glia-α1, -α2, -α3 and DQ8-glia-α1 epitopes according to their genome of origin.

Epitope	Amino acid sequence	A genome	B genome	D genome	Total
DQ2.5-glia-α1	PFPQPQLPY	150	0	104	254
	PFP-PQLPY	0	138	0	138
	P**Y**PQPQLPY	0	0	51	51
	PF**S**QPQLPY	16	0	0	16
	PF**L**QPQLPY	16	0	0	16
	P**Y**PQP**H**LPY	0	0	11	11
	PF**L**-PQLPY	0	10	0	10
	**Q**FP-PQ**Q**PY	0	3	0	3
	PFPQPQL**S**Y	1	0	0	1
	P**S**P-PQLPY	0	1	0	1
	P**Y**P**R**PQLPY	0	0	1	1
	Total	183	152	167	502
DQ2.5-glia-α2	PQPQLPYPQ	0	0	157	157
	P-PQLPYPQ	0	139	0	139
	PQPQLPY**S**Q	123	0	0	123
	PQPQLPY**SH**	27	0	0	27
	**S**QPQLPY**S**Q	16	0	0	16
	**L**QPQLPY**S**Q	16	0	0	16
	PQP**H**LPYPQ	0	0	11	11
	**L**-PQLPYPQ	0	8	0	8
	P-PQ**Q**PYPQ	0	3	0	3
	**L**-PQLPYP**R**	0	2	0	2
	PQPQLPYP**R**	0	0	1	1
	P**R**PQLPYPQ	0	0	1	1
	PQPQL**S**Y**S**Q	1	0	0	1
	Total	183	152	170	505
DQ2.5-glia-α3	FRPQQPYPQ	173	0	93	266
	F**P**PQQPYPQ	0	149	0	149
	FR**Q**QQPYPQ	60	0	0	60
	FRPQQ**S**YPQ	0	0	13	13
	FRPQ**K**PYPQ	4	0	0	4

	**SP**PQQPYPQ	0	2	0	2
	F**Q**PQQPYPQ	1	0	0	1
	F**P**PQQ**S**YPQ	0	1	0	1
	Total	238	152	106	496
DQ8-glia-α1	QGSFRPSQQ	176	2	2	180
	QGSFQ**S**SQQ	0	102	1	103
	QGSFQPSQQ	6	7	48	61
	QG**F**FQPSQQ	0	1	57	58
	Q**V**SFQPSQ**L**	0	40	0	40
	QGSF**R**PSQ**L**	1	0	0	1
	QGSFQPSQ**L**	0	0	1	1
	Total	183	152	109	444

Amino acid residues differing from the canonical epitope are highlighted in bold and deleted residues are represented by a dash.
